# Distribution of valence electrons of the flavin cofactor in NADH-cytochrome *b*_5_ reductase

**DOI:** 10.1038/srep43162

**Published:** 2017-02-22

**Authors:** Kiyofumi Takaba, Kazuki Takeda, Masayuki Kosugi, Taro Tamada, Kunio Miki

**Affiliations:** 1 Department of Chemistry, Graduate School of Science, Kyoto University, Sakyo-ku, Kyoto 606-8502, Japan; 2 Quantum Beam Science Research Directorate, National Institutes for Quantum and Radiological Science and Technology, Tokai-mura, Ibaraki 319-1106, Japan

## Abstract

Flavin compounds such as flavin adenine dinucleotide (FAD), flavin mononucleotide and riboflavin make up the active centers in flavoproteins that facilitate various oxidoreductive processes. The fine structural features of the hydrogens and valence electrons of the flavin molecules in the protein environment are critical to the functions of the flavoproteins. However, information on these features cannot be obtained from conventional protein X-ray analyses at ordinary resolution. Here we report the charge density analysis of a flavoenzyme, NADH-cytochrome *b*_5_ reductase (b5R), at an ultra-high resolution of 0.78 Å. Valence electrons on the FAD cofactor as well as the peptide portion, which are clearly visualized even after the conventional refinement, are analyzed by the multipolar atomic model refinement. The topological analysis for the determined electron density reveals the valence electronic structure of the isoalloxazine ring of FAD and hydrogen-bonding interactions with the protein environment. The tetrahedral electronic distribution around the N5 atom of FAD in b5R is stabilized by hydrogen bonding with C_α_H of Tyr65 and amide-H of Thr66. The hydrogen bonding network leads to His49 composing the cytochrome *b*_5_-binding site *via* non-classical hydrogen bonds between N5 of FAD and C_α_H of Tyr65 and O of Tyr65 and C_β_H of His49.

Prosthetic groups assist in the functions of enzymes by binding as cofactors of protein molecules. Detailed structural information on the prosthetic groups and their environments is indispensable for elucidating the functions of enzymes. However, it is difficult to derive the chemical properties of the prosthetic groups from X-ray structure analysis, since such analysis is conventionally preformed at resolution lower than 1.5 Å, where the fine structural features of protein molecules cannot be deduced. Therefore, precise experimental determination of the structures of cofactors in proteins is desired for a more complete understanding of the mechanism by which the cofactors contribute to the enzymatic function in each protein. Charge-density analyses of X-ray diffraction data at ultra-high resolution can provide details of the electronic structures of cofactors as well as protein environments^1^,^2^,^3^,^4^,^5^,^6^.

Flavin compounds such as flavin adenine dinucleotide (FAD), flavin mononucleotide (FMN) and riboflavin (vitamin B_2_) are utilized as cofactors of proteins for various oxidoreductive processes^7^,^8^,^9^. The isoalloxazine ring is a redox center common to all of these compounds. Two electrons and two protons are accepted and released upon the redox reactions. The redox potential is largely different for each protein due to various interactions with the protein environment. Consequently, a wide variety of types of reactions could be facilitated by the flavin molecules.

NADH-cytochrome *b*_5_ reductase (b5R; EC 1.6.2.2) is a flavoprotein containing one FAD molecule^10^,^11^,^12^,^13^. Its molecular mass is ~34 kDa. A single-spanning transmembrane helix anchors the protein on the inner surface of the endoplasmic reticulum membrane. b5R mediates electron transfer from NADH to cytochrome *b*_5_ (b5). The electrons are utilized in various b5-dependent reactions^14^,^15^,^16^,^17^,^18^. Crystal structures of the soluble domain of b5R have been reported for several species^19^,^20^,^21^,^22^. Although accurate bond lengths and hydrogen positions have been determined in a high resolution crystal structure of porcine b5R^23^, the distribution of electrons critical to the specific properties of the cofactor remains to be elucidated.

In this paper, we report a charge density analysis on the soluble domain of porcine b5R at an ultra-high resolution of 0.78 Å. The valence electronic structures of FAD as well as the protein environment are experimentally determined and analyzed in order to investigate the properties of FAD in b5R.

## Results

### X-ray analysis at ultra-high resolution

**Figure 1:**
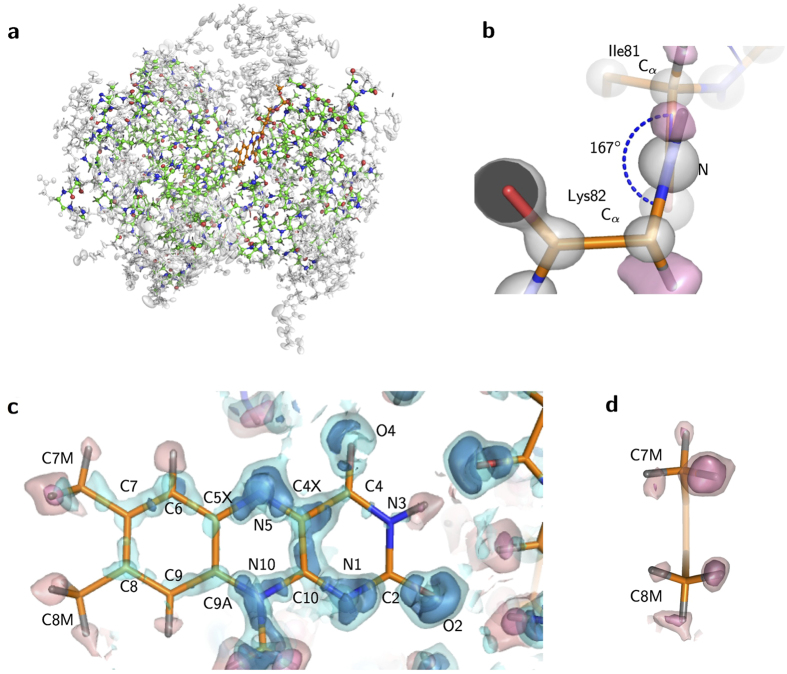
Ultra-high resolution X-ray structure of b5R. (**a**) The ball and stick model of b5R. Atoms refined in the higher order refinement and the subsequent MAM refinement are colored, while other fixed atoms are shown in gray. The ellipsoidal balls represent anisotropic displacement parameters after the MAM refinement at 30% probability. (**b**) Distortion of the peptide bond of Ile81-Lys82 with a *ω* angle of 167°. The hydrogen omit *F*_obs_−*F*_calc_ map is shown as a magenta surface at a contour level of 3.0σ. The 2*F*_obs_−*F*_calc_ map is also shown as a gray surface at a contour level of 5.0σ. (**c**) The residual electron density around FAD. The residual map after the ISAM refinement is shown as cyan and blue surfaces at contour levels of 1.5σ and 2.5σ. Atom names of the isoalloxazine ring are labeled. The hydrogen omit *F*_obs_−*F*_calc_ map is also overlaid as pink and magenta surfaces at the contour levels of 1.5σ and 2.0σ. (**d**) The hydrogen omit *F*_obs_−*F*_calc_ map around the two methyl groups of the isoalloxazine ring of FAD.

An X-ray diffraction data set at 0.78 Å was collected with high-energy X-rays at a synchrotron facility. The structure, which were refined with a conventional procedure^23^, was used as a starting model. Geometric restraints were removed for atoms with low temperature factors (*B*_eq_ < 8.0 Å^2^) in the higher order refinement with the independent spherical atom model (ISAM) parameters just before the multipolar atomic model (MAM) refinement (Fig. 1a). In 21 peptide bonds, the dihedral angles of C_α_−C−N’−C_α_’ (*ω*) are twisted more than 10° from the planar configuration with an angle of 180° (Fig. 1b), which is usually assumed and restrained in protein structural analyses^24^. Residual electron densities are clearly observed around main chain atoms without any averaging treatment (Supplementary Fig. 1a). These densities can be interpreted as bonding or lone pair electrons according to their locations. In addition, residual densities are also observed at many side chains (Supplementary Fig. 1b and c) and the isoalloxazine ring of the FAD cofactor (Fig. 1c).

The residual densities were analyzed with MAM in order to obtain the charge-density information. The residual densities were decreased by the refinement (Supplementary Fig. 2). The *R*_work_ and *R*_free_ factors dropped from 12.6% and 14.4% to 12.2% and 14.0%, respectively (Table 1). The final model contained 51 hydrogen atoms of water molecules in addition to 76% of protein hydrogen atoms. Multiple conformations were observed for the 48 residues.

All hydrogen atoms in the isoalloxazine and ribityl groups were clearly observed in the hydrogen omit map (Fig. 1c). Rotameric structures of the two methyl group of the isoalloxazine ring could be experimentally determined (Fig. 1d). In addition, no electron densities corresponding to the HN10 and HN5 hydrogen atoms were observed. This demonstrated that the FAD cofactor in the crystal is in the oxidized state. On the other hand, the electron density for the HN3 hydrogen atom was unambiguously visualized in the hydrogen omit map (Fig. 1c).

**Figure 2:**
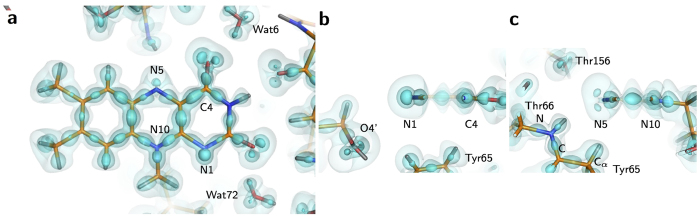
Valence electron distributions of FAD. (**a**) The deformation map around the isoalloxazine ring of FAD. The cyan surfaces represent the electron density at contour levels of +0.01, +0.2 and +0.5 e/Å^3^, respectively. The view is the same as in Fig. 1c. (**b**) The cross section along the N1-C4 line. (**c**) The cross section along the N5-N10 line.

The MAM analysis provides more accurate atomic positions uninfluenced by the valence electrons. The root mean square deviation (rmsd) between the SHELX and the final MAM structure is 0.01 Å for all the atoms. The deformation map clearly captures the features of the electronic structure of the FAD molecule (Fig. 2a), while such features are already visualized in the residual map of the ISAM model (Fig. 1c). Distributions of lone pair electrons are observed as expected for around N1, O2 and O4 atoms (Fig. 2a and b). However, those for around the N5 atom have out-of-plane components (Fig. 2c and Supplementary Fig. 3), while the atom is assigned as being in the *sp*^2^ hybridization state according to the structural formula of FAD.

### Topological analyses for covalent bonds of FAD

**Figure 3:**
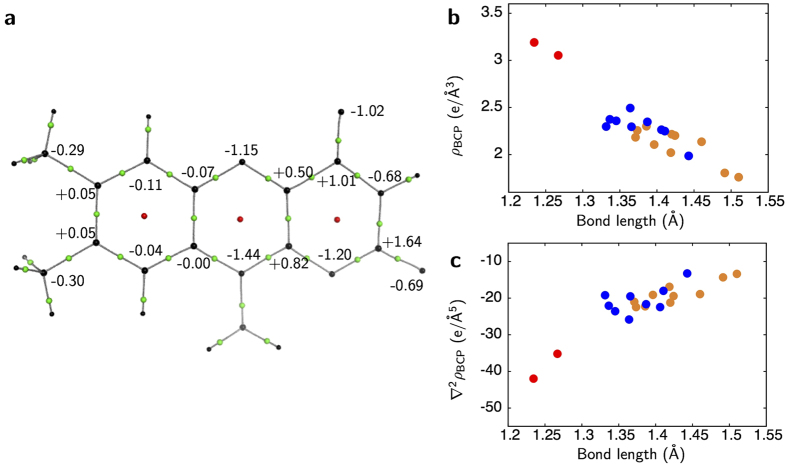
Properties of the covalent bond for FAD. (**a**) The molecular graph of the isoalloxazine ring. BCPs and ring critical points (RCPs) are shown as green and red spheres. Bond paths are represented as gray curves. The atomic charges are indicated in the proximity of each atom. The view is the same as in Fig. 1c. (**b**) Dependence between bond length and *ρ*_BCP_. Filled circles in yellow, blue and red are for C−C, N−C and O−C bonds, respectively. (**c**) Dependence between bond length and ∇^2^*ρ*_BCP_.

The topological analysis of the charge-density with the Atoms in Molecules (AIM) theory can extract information about electronic structures^25^,^26^. The Laplacian and gradient maps reveal bond paths and atomic boundaries (Supplementary Fig. 4). The atomic charges with consideration of the atomic boundaries are derived for the isoalloxazine ring (Fig. 3a and Supplementary Table 1). The nitrogen and oxygen atoms have negative charges, and most of the carbon atoms have smaller positive or negative charges, as expected. However, some carbon atoms, such as C2, C4 and C10, which bind more than two nitrogen or oxygen atoms, have larger positive charges.

Bond critical points (BCPs) have the minimum *ρ* value along the bond path, while they have the maximum *ρ* value on the cross section of the bond path. The *ρ* value (*ρ*_BCP_) and Laplacian of *ρ* (∇^2^*ρ*_BCP_) at BCP represent properties of the chemical bonding. BCPs can be detected with the topological analysis^25^. A BCP is observed for each covalent bond (Fig. 3a and Supplementary Table 2). The *ρ*_BCP_ values of covalent bonding in the isoalloxazine ring are plotted against the bond length (Fig. 3b and Supplementary Table 2). All data in the plot approximately lie on an almost straight line. In addition, the ∇^2^*ρ*_BCP_ values for the C−C, C−N and C−O bonds are negative, and have significant correlation with the bond lengths (Fig. 3c and Supplementary Table 2). Covalent bond orders *n*_topo_ were derived from the topological analysis (Supplementary Table 2). For the C−C bonds in the isoalloxazine ring, the average *n*_topo_ value is 1.39. This value is reasonable as the conjugated double bonds^27^. However, only the C4−C4X bond has a relatively low value of 1.22. This indicates that the bond has a lower conjugated nature than the others. The average value for the C−N bonds is 1.15. This indicates nearly single bonding, while the conjugated double bonds are expected from the chemical formula. The *n*_topo_ values for the two C−O bonds are 1.6 and 1.7, indicating double bonding^28^.

### Topological analyses for hydrogen bonds

**Figure 4:**
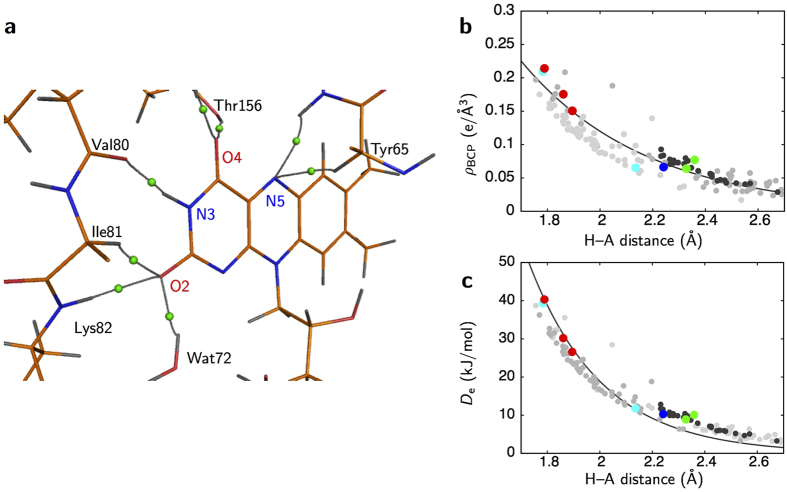
Interactions between FAD and the protein environment. (**a**) The bond paths of hydrogen bonding around the isoalloxazine ring. BCPs of the hydrogen bonding are represented as green spheres. (**b**) Dependence between 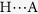 distance and *ρ*_BCP_ for hydrogen bonding. Filled circles in cyan, blue, red and green are for NH
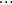
O, NH
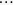
N, OH
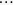
O and CH
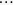
N/O bonds, respectively. A relationship derived from small molecules^29^ is overlaid as a solid curve in gray. Small gray circles represent the NH
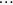
O, NH
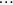
N and OH···O bonds in the main chain. Dark-gray circles also represent the C_α_H
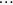
N/O bonds. (**c**) Dependence between the 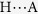 distance and dissociation energy *D*_e_.

The bond paths for hydrogen bonding are slightly curved (Fig. 4a), while those for covalent bonding are almost straight. Bond paths and BCPs are observed for classical hydrogen bonding between atoms of FAD and environmental peptides such as N5 and NH of Thr66, HN3 and O of Val80, O2 and NH of Lys82, and O4 and O_γ_H of Thr156. Bond paths are also detected between O2 and Wat72, and O4 and Wat6. In addition, bond paths for non-classical hydrogen bonding between N5 and C_α_H of Tyr65, and O2 and C_α_H of Ile81 are detected. On the other hand, the N1 atom, which is one of the hydride addition sites along with the N5 atom, has no bond paths for hydrogen bonding.

The *ρ*_BCP_ *vs*. hydrogen-to-acceptor (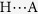) distance plot for all hydrogen bonding between FAD and the protein environment shows a curved relationship (Fig. 4b and Supplementary Table 3), as in the case for small molecules^29^. This relationship is also true for the two non-classical hydrogen bonding interactions. Both ∇^2^*ρ*_BCP_ and the total electron energy densities *H*_BCP_ values are positive for all the hydrogen bonding. This indicates that the bonds are noncovalent closed-shell interactions, as expected in the case of the normal hydrogen bonding^30^. The dissociation energy *D*_e_ can also be estimated from the *ρ*_BCP_ and ∇^2^*ρ*_BCP_ values^31^. The *D*_e_ values for almost all hydrogen bonding in the protein are in the range from ~10 to 40 kJ/mol (Supplementary Table 3), which is typical for normal hydrogen bonding^32^. The *D*_e_ values also have a significant correlation with 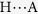 distance (Fig. 4c). For hydrogen bonding around FAD, the *D*_e_ values and correlation with 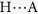 distances are in line with other hydrogen bonds in the protein (Fig. 4c).

## Discussion

**Figure 5:**
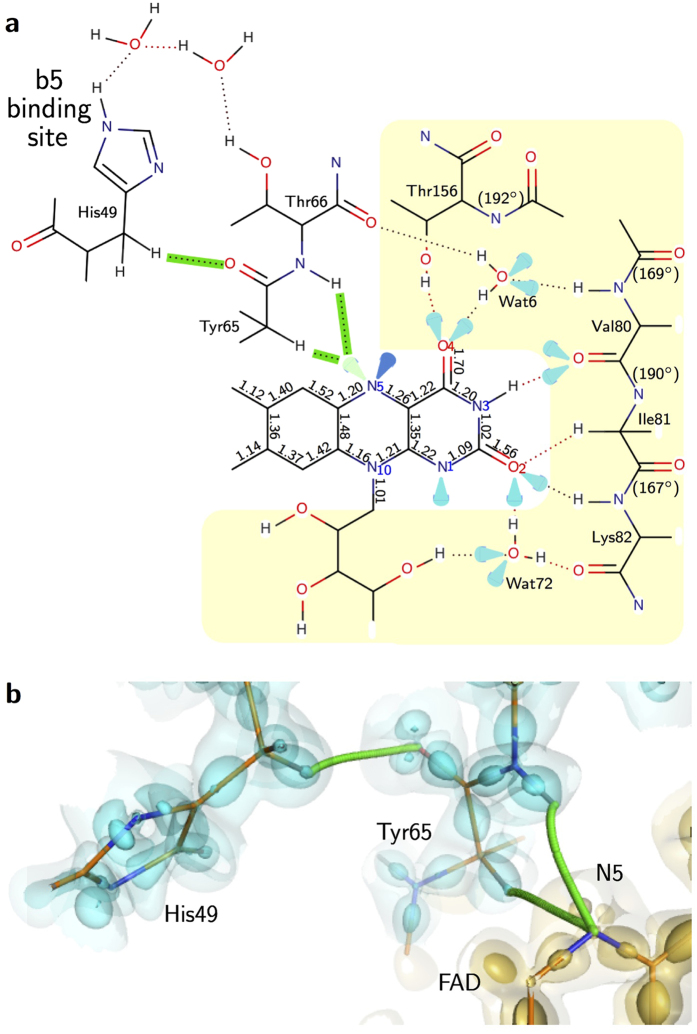
Interactions around FAD and the possible pathways of electron and proton. (**a**) Schematic representation of the electronic structure of FAD and the surrounding residues. The lone pair electrons are represented as drop marks. The interactions detected in the topological analysis are shown as dotted lines. The bond orders are indicated in the proximity of each bond. The *ω* angles for the peptide bonds with larger distortion (|*ω* − 180| > 10) are also indicated in the parenthesis. The hydrogen bonds for electron transfer are highlighted in green color. (**b**) The electron pathways along the hydrogen-bond paths. The cyan and yellow surfaces represent the deformation electron densities as in Fig. 2. Hydrogen bond paths are represented as green curves.

The accurate charge density analysis and subsequent topological analysis for b5R provide various indexes of the electronic properties, such as the atomic charge, electronic distribution and bond order. The *ρ*_BCP_ *vs*. bond length plot shows a negative correlation as in the case of small molecules. In addition, the ∇^2^ *ρ*_BCP_ *vs*. bond length plot has a similar trend with those for small molecules^26^. Therefore, we can discuss the electronic structure of FAD and hydrogen bonding interactions in b5R (Fig. 5a) with high reliability. On the other hand, some of the results are unexpected. For example, the bond orders for the C−N bonds in the isoalloxazine ring are only about 1.15 on average, indicating single bonds. However, this value is reasonable according to the experimental and theoretical results for small molecules^27^,^33^.

Some unexpected results are also derived for hydrogen bonding. The 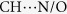 -type hydrogen bonding significantly contributes to the interaction between FAD and the protein environment. In fact, non-classical hydrogen bonding has been detected in some proteins^6^,^34^,^35^ as well as many small compounds^36^. The importance of non-classical hydrogen bonding has been mainly pointed out for the stability of macromolecules. Also in b5R, sufficient number of the bonding between main chains are observed (Fig. 4b and c). They are mainly formed between strands as reported^34^. The energies of non-classical hydrogen bonds are smaller than those of classical bonds. This topic should be investigated for a wider range of proteins in both experimental and theoretical studies in order to extract essential features by comparison. The *H*_BCP_ values are positive for hydrogen bonding between FAD and the protein environment in b5R, while those in cholesterol oxidase are negative^6^. This may be due to the contribution of the resonance-assisted effect^30^ in cholesterol oxidase.

The most unexpected feature of FAD in b5R is the lone pair distributions around the N5 atom. The tetrahedral electronic distribution around N5 seems to be stabilized by hydrogen binding with C_α_H of Tyr65 and amide-H of Thr66 (Figs 4a and 5a). In addition, no atoms appropriate for the hydrogen bonding are located on the same or nearly the same plane as the isoalloxazine ring, in contrast to the cases of N1, O2 and O4 atoms having normal electronic distributions. This implies that the electronic distributions of the flavin cofactor can be easily affected by the interactions with the respective protein environments. On the other hand, we have to worry that the electronic distribution around N5 is an experimental and/or analytical artifact. However, we believe that the result is relevant for two reasons as follows. First, the radiation damages could be suppressed by the collection of data at low accumulated doses (1.8 × 10^5^ Gy for the first data set (Data I) and 0.9 × 10^5^ Gy for the second data set (Data II)) at 40 K. The values were smaller than the Henderson limit of 2.0 × 10^7^ Gy applied to ordinal measurements around 100 K by more than two orders of magnitude^37^. Furthermore, it has been reported that the photoreduction is suppressed significantly at 10–40 K^38^,^39^. The second reason is that the valence electrons were clearly observed for both Data I and II, even in the residual maps of the ISAM refinements (Supplementary Figs 3a and 5a) as well as the deformation maps of the MAM refinements (Supplementary Figs 3b and 5c). These multiple results enhance the validity of this study. Our results may also be significant even if the photoreduction was not completely suppressed as planned. In such a case, the structure may not represent the oxidized form but the red semiquinone form, which plays an important role in the redox cycle of b5R.

It should be noted that the hydrogen bonding network originated from the valence electrons of the N5 atom leads to His49 through Thr66, while the N1 atom forms no bond path of hydrogen bonding with the protein environment. His49 is one of residues composing the b5-binding site^23^,^40^,^41^,^42^. The non-classical bond between O of Tyr65 and C_β_H of His49 realizes a shorter path for electron transfer than paths along only the classical bonds (Fig. 5a and b). Furthermore, the N3 and O2 atoms at the hydrophilic side of the isoalloxazine ring interact with the distorted peptide bonds of successive residues from Leu79 to Lys82 (Fig. 5a). The O4 atom also interacts with the distorted peptide bond between Ile155 and Thr156, which has a large deviated *ω* angle of 192°. The electron transfer toward these directions may be blocked by the decreased delocalization properties of the distorted peptide bonds, while peptides can act as a conductor for electron transfer^43^. Therefore, our results plausibly imply that the directivity of the electron transfer is realized by the protein environment in addition to the nature of FAD itself.

The charge-density analysis in this study revealed the fine features of FAD in the protein environment based on high-quality X-ray data. In combination with other experimental and computational results, these findings will make a unique and significant contribution to our understanding of the electronic structural basis of functional mechanisms of b5R.

## Methods

### Preparation of crystals

The recombinant soluble domain consisting of 272 residues of porcine b5R was expressed in *Escherichia coli* and purified as described previously^23^,^41^. Crystallization experiments were performed by the hanging drop vapor diffusion method under the same conditions as described previously^23^,^44^. Briefly, 5 μL of protein solution containing 40 mg/mL b5R and 10 mM potassium phosphate (pH 7.0) were equilibrated against 5 μL of precipitant solution containing 9–12% (w/v) PEG 4,000, 100 mM potassium phosphate (pH 7.7) and 5 mM dithiothreitol (DTT) at 293 K. Crystals with a typical size of 1.0 × 0.3 × 0.2 mm^3^ were obtained within 1 week.

### X-ray diffraction experiment

The crystals were flash-frozen with a helium-gas stream of 40 K after soaking in a solution containing 20% (v/v) glycerol, 10% (w/v) PEG 4,000, 10 mM potassium phosphate (pH 7.0) and 5 mM DTT. Diffraction data were measured at the BL41XU beamline of SPring-8. The wavelength of X-rays and the beam size were set to 0.65 Å and 30 × 30 μm^2^, respectively. The diffraction intensities were recorded with a Rayonix MX-225HE CCD detector. The helical data-collection method^45^ was used to collect high-resolution data. Low-resolution data were collected from a non-irradiated position of the crystal. The crystal-to-detector distances were set to 70 and 250 mm for high and low resolution data collections. The diffraction data were integrated, scaled and merged with the HKL2000 program package^46^. The crystallographic statistics are listed in Table 1. The maximum dose for each irradiated position was estimated to be 1.8 × 10^5^ Gy with the RADDOSE program^47^. Another complete data set (Data II) was also collected from a different crystal (Supplementary Table 4). The resolution and maximum dose for Data II were 0.8 Å and 0.9 × 10^5^ Gy, respectively.

### Structure refinement with the ISAM

The previously reported structure of porcine b5R (PDB-ID, 1NDH)^48^ was used as an initial model in the molecular replacement method with the MOLREP program^49^. Structure refinement with an independent spherical atom model (ISAM) was carried out with the CNS and SHELXL programs, successively^50^,^51^. All non-hydrogen atoms were refined with anisotropic *B* factors. Hydrogen atoms were added to the model by SHELXL as “riding-hydrogens”, except for those riding on residues that have multiple conformations and the adenosine moiety of FAD. The *R*_work_ and *R*_free_ factors at the final SHELXL refinement were 12.6% and 14.4%, respectively. After the refinement with SHELXL, an additional scaling was performed in the same manner as described previously^2^ in order to correct the resolution-dependence in the absorption of the CCD detector. The 51 hydrogen atoms of water molecules were added as the riding model on the strong (>2σ) peaks in the residual map at last, and any atoms are not added or removed in the following MAM refinement step.

### Charge-density analysis with the MAM

The MAM is expressed as follows^52^:


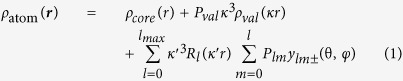


The first two terms describe the spherically symmetric core and valence electron densities, and the third one the non-spherical distribution of the valence electrons. *P*_val_ and *P*_lm_ are population coefficients. The *R*_l_ are Slater-type radial functions, and the *y*_lm±_ are real spherical harmonic angular functions. Multi-conformational residues, waters without two hydrogens and atoms with high temperature factors (*B*_eq_ > 8 Å^2^) were not selected for the MAM refinements with the MOPRO program^53^. 55.3% of all atoms, including the isoalloxazine and ribityl groups of FAD and 13 water molecules, were employed for the refinements. Prior to the MAM refinement, higher-order refinement was performed for the selected atoms using data in the resolution range of 1.0–0.78 Å without geometric restraints. Then, the initial values of the multipole parameters were transferred from the experimental library multipolar atom model (ELMAM)^54^. The initial values for FAD were prepared from the experimental values for NAD^+^, adenine, cytosine, thiouracil and ethionamide^55^,^56^,^57^,^58^. Positions of H atoms were changed to the standard geometry from neutron diffraction analyses^59^. The *P*_val_ and *P*_lm_ values of non-water and non-hydrogen atoms were refined in the MAM refinement, while *κ* and *κ*′ were fixed to the initial values. The *P*_val_ and *P*_lm_ values were constrained based on the chemical similarities. To refine the MAM parameters of N5 in FAD, a grid search was performed by using parameter sets describing *sp*^2^, *sp*^3^ -like and some intermediate cases. The most appropriate result was obtained at *sp*^2^:*sp*^3^ = 0.2:0.8, judging from the density (Supplementary Fig. 3). The *R*_work_ and *R*_free_ factors at the final MAM refinement were converged to 12.2% and 14.0%, respectively. Data II was analyzed in the same manner as described above for Data I. Refinement and model statistics for Data I and Data II are listed in Table 1 and Supplementary Table 4, respectively. The deformation maps were calculated by using equation (2)^3^.


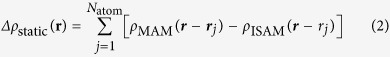


The two-dimensional contour maps were prepared using the VMoPro program^53^ and the three-dimensional figures were prepared using PyMOL^60^.

Topological analysis based on the AIM theory was performed with the VMoPro program. The atomic charges by the AIM theory were calculated with the BADER program^61^. The bond orders *n*_topo_ were calculated according to a method described previously^27^. The total electron energy density *H*_BCP_ and dissociation energy *D*_e_ of hydrogen bonding were calculated according to the equations:


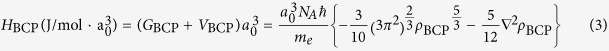



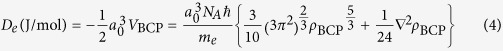


where *G*_BCP_ and *V*_BCP_ are the kinetic and potential energy densities at BCPs^62^.

## Additional Information

**Accession codes**: The coordinates and structure factors for Data I and II have been deposited in the Protein Data Bank under accession numbers 5GV8 and 5GV7, respectively.

**How to cite this article:** Takaba, K. *et al*. Distribution of valence electrons of the flavin cofactor in NADH-cytochrome *b*_5_ reductase. *Sci. Rep.* **7**, 43162; doi: 10.1038/srep43162 (2017).

**Publisher's note:** Springer Nature remains neutral with regard to jurisdictional claims in published maps and institutional affiliations.

## Supplementary Material

Supplementary Information

## Figures and Tables

**Table 1:** Crystallographic and refinement statistics.

	Data I
**Data collection**	
Space group	*P*2_1_2_1_2_1_
Cell dimensions	
*a, b, c* (Å)	48.480, 72.108, 84.908
Resolution (Å)	31.9–2.0 (low resolution) 10.0–0.78 (0.79–0.78)^a^ (high resolution)
*R*_merge_^b^(%)	7.1 (118.6)^a^
*I*/σ*I*	28.8 (1.1)^a^
Completeness (%)	99.1 (89.5)^a^
Redundancy	6.7 (4.7)^a^
CC_1/2_ (%)	(51.9)^a^
**Refinement**	
Resolution (Å)	31.9−0.78
No. reflections	332946
*R*_work_^c^/*R*_free_^d^ (%) (ISAM/SHELX)	12.6/14.4
*R*_work_^c^/*R*_free_^d^ (%) (MAM/MOPRO)	12.2/14.0
No. non-H atoms	
Protein	2390
Ligand/ion	90
Water	608
No. H atoms	
Protein	2012
Ligand/ion	20
Water	51
No. multipole parameters	24930

^a^Highest resolution shell is shown in parentheses.

^b^*R*_merge_ = Σ_hkl_Σ_i_| *I*_hkl,i_ − 〈*I*_hkl_〉|/Σ_hkl_Σ_i_ *I*_hkl,i_.

^c^*R*_work_ = Σ_hkl_||*F*_obs_| − |*F*_calc_||/Σ_hkl_|*F*_obs_|.

^d^*R*_free_ was calculated by using 5% of the reflections that were not included in the refinement as a test set.
