# Supplementary material for: Distribution of valence electrons of the flavin cofactor in NADH-cytochrome b5 reductase
Source: Sci Rep. 2017 Feb 22;7:43162. doi: 10.1038/srep43162 (PMC5320556; doi:10.1038/srep43162)
Supplement: Supplementary Information [file srep43162-s1.pdf]

Supplementary Information

Distribution of valence electrons of the flavin cofactor in NADH-cytochrome *b*$_5$ reductase

Kiyofumi Takaba[1,*], Kazuki Takeda[1,*], Masayuki Kosugi[1], Taro Tamada[2] and Kunio Miki[1,**]

[1]Department of Chemistry, Graduate School of Science, Kyoto University, Sakyo-ku, Kyoto 606-8502, Japan.

[2]Quantum Beam Science Research Directorate, National Institutes for Quantum and Radiological Science and Technology, Tokai-mura, Ibaraki 319-1195, Japan.

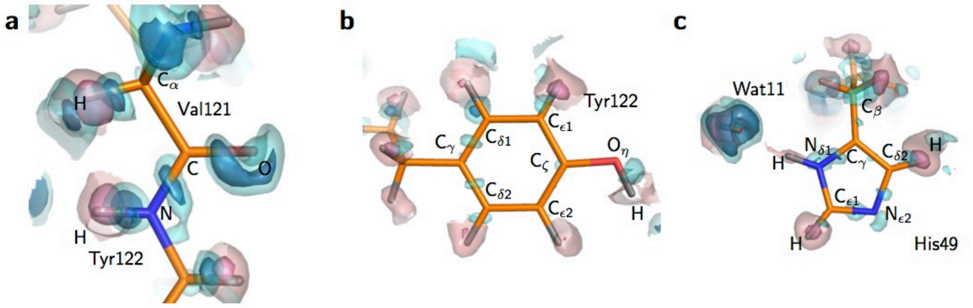

Supplementary Figure 1 | Residual electron density after the ISAM refinement

(**a**) The residual electron density around the peptide bond of Val121-Tyr122. The residual F_{obs}–F_{calc} map is shown as cyan and blue surfaces at contour levels of 1.75σ and 2.5σ. The hydrogen omit F_{obs}–F_{calc} map is overlaid as pink and magenta surfaces at contour levels of 1.5σ and 3.0σ. (**b**) The residual electron density around the side chain of Tyr122. (**c**) The residual electron density around the side chain of His49.

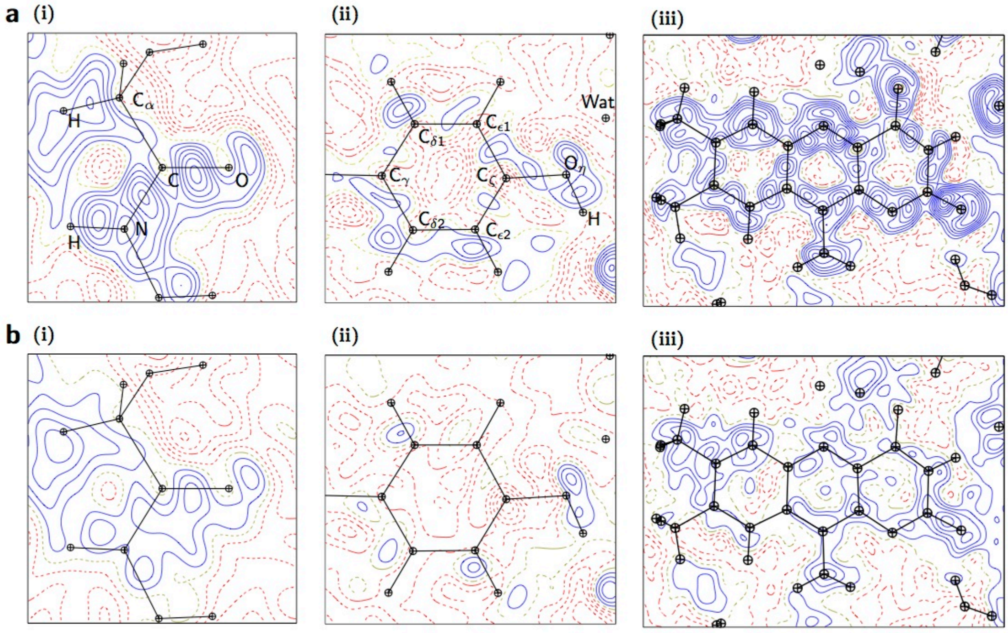

Supplementary Figure 2 | Contour maps of the residual electron density for each refinement step

(**a**) The residual electron densities after the ISAM refinement are shown at the interval of 0.05 e/Å^3. (i) The peptide bond between Val121 and Tyr122. (ii) The side chain of Tyr122. (iii) The isoalloxazine ring of FAD. (**b**) The residual electron densities after the MAM refinement.

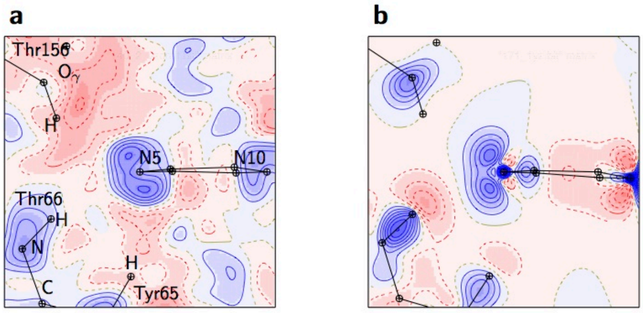

Supplementary Figure 3 | Electron density maps around the N5 atom of FAD

(a) The residual electron density after the ISAM refinement is shown at the interval of 0.05 e/Å^3. The view is the same as in Fig. 2c. (**b**) The static deformation map.

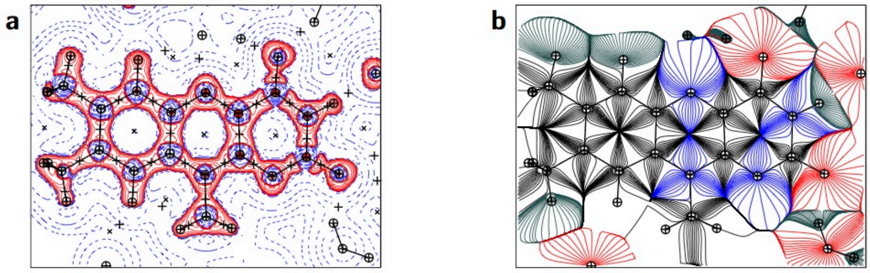

Supplementary Figure 4 | Topological analysis

(**a**) The Laplacian $\nabla^2\rho$ map around the isoalloxazine ring. The view is the same as in Fig. 1c. The contour interval is 0.05 e/Å^5. Red solid and blue dashed lines represent positive and negative levels, respectively. BCPs and RCPs are represented as "+" and "×", respectively. (**b**) The gradient vector field.

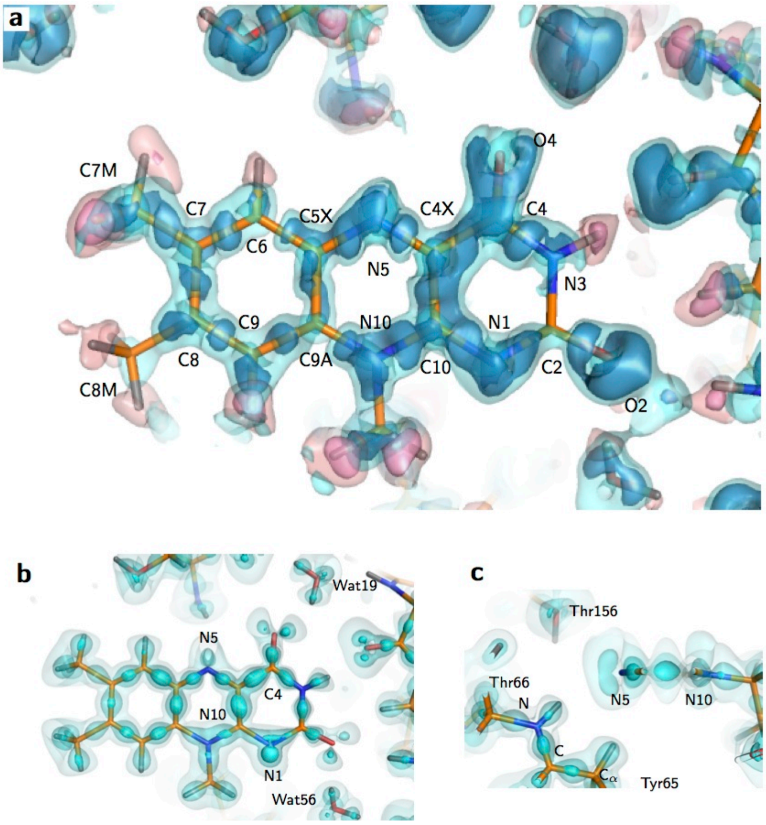

Supplementary Figure 5 | Electron densities for Data II

(**a**) The residual electron density around the isoalloxazine ring of FAD. The F_{obs}–F_{calc} map after the ISAM refinement is shown at contour levels of 1.5σ (cyan) and 2.5σ (blue). The hydrogen omit F_{obs}–F_{calc} maps are also overlaid as pink and magenta surfaces at the contour levels of 2.0σ and 3.0σ. (**b**) The deformation map around the isoalloxazine ring of FAD. The cyan surfaces represent the electron density at contour levels of +0.01, +0.2 and +0.5 e/Å^3, respectively. (**c**) A close-up view of the deformation map for the N5 atom.

Supplementary Table 1. Atomic charges

Atom	Charge
O4	-1.02
C4	+1.01
N3	-0.68
HN3	+0.53
C2	+1.64
O2	-0.69
N1	-1.20
C10	+0.82
C4X	+0.50
N5	-1.15
C5X	-0.07
C6	-0.11
H6	+0.31
C7	+0.05
C7M	-0.29
HM71	+0.22
HM72	+0.21
HM73	+0.22
C8	+0.05
C8M	-0.30
HM81	+0.22
HM82	+0.21
HM83	+0.22
C9	-0.04
H9	+0.24
C9A	-0.00
N10	-1.44
C1’	+0.10
H1’1	+0.23
H1’2	+0.24
Total	+0.04

Supplementary Table 2. Bond properties of FAD

	length (Å)	ρ_{BCP}(e/Å^3)	$\nabla^2\rho_{BCP}$(e/Å^5)	n_{topo}
C4–C4X	1.460(8)[a]	2.14	-18.9	1.22
C10–C4X	1.420(8)	2.22	-21.2	1.35
C5X–C6	1.386(8)	2.30	-22.2	1.52
C5X–C9A	1.424(8)	2.20	-19.4	1.48
C6–C7	1.371(8)	2.18	-21.0	1.40
C7–C7M	1.510(9)	1.76	-13.4	1.12
C7–C8	1.419(9)	2.02	-16.9	1.36
C8–C8M	1.491(9)	1.81	-14.3	1.14
C8–C9	1.396(9)	2.11	-19.1	1.37
C9–C9A	1.374(8)	2.26	-22.5	1.42
C4–N3	1.364(7)	2.49	-25.8	1.20
N3–C2	1.406(8)	2.26	-22.5	1.02
C2–N1	1.345(8)	2.36	-23.6	1.09
N1–C10	1.332(7)	2.30	-19.2	1.22
C10–N10	1.387(7)	2.35	-21.7	1.21
C4X–N5	1.340(7)	2.37	-22.1	1.26
N5–C5X	1.370(7)	2.30	-19.5	1.20
C9A–N10	1.411(7)	2.25	-18.0	1.16
N10–C1’	1.443(8)	1.99	-13.3	1.01
O4–C4	1.235(7)	3.19	-42.0	1.70
C2–O2	1.267(7)	3.05	-35.2	1.56

[a]Values in parentheses are estimated standard deviations derived from the full-matrix least squares refinement.

Supplementary Table 3. Properties of hydrogen bonding around FAD

Donor	Acceptor	$d_{\text{H-A}}$ (Å)	ρ_{BCP}(e/Å^3)	$\nabla^2\rho_{BCP}$ (e/Å^5)	H_{BCP}(kJ/mol·a$_0^3$)[b]	D_e (kJ/mol)
C_α-Tyr65	N5-FAD	2.36(7)[a]	0.077	1.26	7.06	10.09
N-Thr66	N5-FAD	2.24(8)	0.066	1.51	10.33	10.28
N3-FAD	O-Val80	1.78(9)	0.210	3.57	9.29	39.29
C_α-Ile81	O2-FAD	2.33(8)	0.064	1.25	8.19	8.89
N-Lys82	O2-FAD	2.14(9)	0.065	1.86	13.60	11.77
O_γ-Thr156	O4-FAD	1.89(10)	0.151	2.92	13.13	26.58
Wat6	O4-FAD	1.86(10)	0.176	2.86	8.81	30.20
Wat72	O2-FAD	1.79(10)	0.214	3.59	8.67	40.32

[a]Values in parentheses are estimated standard deviations derived from the full-matrix least squares refinement.

[b]a_0 is the Bohr radius.

Supplementary Table 4 | Crystallographic and refinement statistics for Data II

	Data II
**Data collection**	
Space group	$P2_12_12_1$
Cell dimensions	
a, *b*, *c* (Å)	48.664, 72.177, 85.209
Resolution (Å)	29.3-2.0 (low resolution),
	10.0-0.80 (0.81-0.80)[a] (high resolution)
R_{merge}[b] (%)	6.7 (185.7)[a]
$I/\sigma I$	31.6 (1.3)[a]
Completeness (%)	99.9 (99.9)[a]
Redundancy	7.5 (6.8)[a]
$CC_{1/2}$ (%)	(41.4)[a]
**Refinement**	
Resolution (Å)	29.3-0.80
No. reflections	314475
R_{work}[c]/ R_{free}[d](%) (ISAM/SHELX)	13.8/15.8
R_{work}[c]/ R_{free}[d] (%) (MAM/MOPRO)	12.2/14.4
No. non-H atoms	
Protein	2427
Ligand/ion	90
Water	620
No. H atoms	
Protein	2200
Ligand/ion	19
Water	31
No. multipole parameters	23103

[a]Highest resolution shell is shown in parentheses.

[b]$R_{merge} = \Sigma_{hkl}\Sigma_i| I_{hkl,i}-<I_{hkl}>|/\Sigma_{hkl}\Sigma_i I_{hkl,i}$.

[c]$R_{work} = \Sigma_{hkl}||F_{obs}|-|F_{calc}||/\Sigma_{hkl}|F_{obs}|$.

[d]R_{free} was calculated by using 5% of the reflections that were not included in the refinement as a test set.
